# High Capacity Nano-Sized Carbon Spheres for Lithium-Ion Battery Anode Materials

**DOI:** 10.3390/polym11040645

**Published:** 2019-04-09

**Authors:** Youliang Wang, Guoyun Yu, Xiujuan Chen, Ansong Wang

**Affiliations:** 1 School of Mechanical and Electrical Engineering, Lanzhou University of Technology, Lanzhou 730050, China; wangyouliang20@163.com; 2 State Key Laboratory of Advanced Processing and Recycling of Non-ferrous Metals, Lanzhou University of Technology, Lanzhou 730050, China; ygywjj@126.com (G.Y.); 17361558956@163.com (A.W.)

**Keywords:** carbon sphere, hydrothermal method, anode materials, lithium-ion batteries

## Abstract

A one-step hydrothermal method is reported for synthesizing carbon spheres (Cs) with sucrose as the carbon resource for the anode materials in lithium-ion batteries (LIBs). Firstly, the influences of synthesis temperature and time on particle size and the morphology of the Cs were researched. Then, modified carbon spheres (MCs) were synthesized with some surfactants, such as hexadecyl trimethyl ammonium bromide (CTAB) and polyvinyl alcohol (PVA). Finally, nano-sized MCs with an average diameter of 70 nm, owning the smooth surface and uniform spherical morphology systematically investigated by X-ray diffraction (XRD), scanning electron microscope (SEM), and transmission electron microscope (TEM). The outstanding performances of nano-sized MCs synthesized with PVA were demonstrated as anode materials in LIBs. The higher initial discharge capacity of 1180 mAhg^−1^ and the excellent discharge capacity of 470 mAhg^−1^ were obtained respectively at 100 mAg^−1^ (0.27 C) over 50 cycles. The nano-sized MCs has also shown remarkable performance of rate capability of 284.6 mAhg^−1^ at 1.5 C. In addition, the cycling reversibility of the nano-sized MCs is more stable than that of the sub-micron sized MCs modified with CTAB and no surfactant respectively.

## 1. Introduction

Since the 21st century, with the development of the social economic, many countries all over the world emphasize on the problem of the energy resources, which have tight relationships with social development, stability and national securities. With the increasingly serious environmental problems and the reduction of non-renewable resources, the development and application of new-energy materials become an urgent issue. Therefore, these problems have drawn the researchers’ attention and are pushing researchers to develop the clean, effective and sustainable technologies to supply and store energy such as lithium–sulfur batteries [1], lithium–air batteries [2], zinc–air batteries [3,4], sodium–ion batteries [5] and lithium–ion batteries (LIBs), and so on. 

In recent years, LIBs as an important energy storage device have been widely applied in our daily lives, due to their excellent characteristics, such as high capacity, high power density, long lifespan, high energy, and low cost. The properties of LIBs have closely linked to cathode electrode materials, as well as anode electrode materials. For a long-term investigation, LiCoO_2_, LiMnO_2_, LiFePO_4_, and V_2_O_5_ were chosen as the cathode materials, due to their advanced properties. As for the anode electrode materials, the researchers focused on carbonaceous materials, mental oxides, polymers, and nano-alloys. Since Sony Corporation used petroleum coke as anode electrode materials for LIBs in 1990, more attention has been paid to carbonaceous materials [6]. Up to now, carbonaceous materials are mostly widely applied among the all kinds of anode materials, owing to their characteristics, such as good conductivity, low cost, and a high voltage plateau. Nevertheless, there are still a number of problems for the anode electrode materials of LIBs, such as severe irreversible capacity loss during the initial charge and discharge process, poor cycle stability, and a nonideal high rate of charge and discharge performance, due to anisotropy during the process of lithium intercalation [7]. Wang. et al. [8] studied the behaviors of LIBs using monodispersed hard carbon spherules as anode electrodes that delivered a reversible capacity of about 430 mAhg^−1^ after 10 cycles. Wang. et al. [9] reported that spherical carbon from porous starch as anode materials, and the carbon spheres that they had prepared maintained a good capacity at 513 mAhg^−1^ after 50 cycles. 

A spherical structure is supposed to be an efficient method for shortening the transport length, for providing abundant active sites and space for a chemical reaction, and for buffering the volume change during insertion and extraction. However, based on current research, carbon spheres (Cs) have common problems, such as a wide distribution of particle sizes, and a poor dispersity, which can lead to terrible cross-linking reactions. When Cs are applied as the anode materials in LIBs, severe cracks can be found on the surface of the Cs after cycling. Furthermore, further improvement of the performance of the Cs anode materials are necessary for application.

In this work, sucrose was selected as the precursor, and then the Cs were synthesized through a facile one-step hydrothermal method. Afterwards the effects of the experimental conditions given in this study on the morphology and diameters of the Cs were explored to discuss. The produced Cs exhibited better dispersity and smaller particle sizes than those reported previously. Finally, the modified carbon spheres (MCs) were synthesized by the addition of surfactants to reduce the size of Cs that the nano-sized, submicron-sized and micron-sized MCs were successfully prepared. The synthesized nano-sized MCs exhibited enhanced cycle performance and lithium storage capacity, compared with those of sub-micron sizes.

## 2. Experimental 

### 2.1. Synthesis of Carbon Spheres (Cs)

The Cs were prepared by a simple one-step hydrothermal synthesis, as described in a previous report [10]. In a typical procedure, the appropriate amount of sucrose (Tianjin Guangfu Technology development CO., LTD, Tianjin, China) was added into 60 mL distilled water under vigorous magnetic stirring for 30 min, to form a 0.3 M sucrose aqueous solution. Then, the obtained solution was transferred into a 100 mL Teflon-lined stainless steel autoclave, and kept at a constant temperature in the range of 140–220 °C, increased every 20 °C: 140 °C, 160 °C, 180 °C, 200 °C and 220 °C for 4 h. The obtained products were labelled as S-0, S-1, S-2, S-3, and S-4 respectively. After that, the 0.3 M sucrose aqueous solution was remade as described above. The solution obtained was transferred into a 100 mL Teflon-lined stainless steel autoclave, and for different time periods of 4 h, 5, 6, 8 and 12 h at 160 °C. The obtained Cs were named as S-5, S-6, S-7, S-8, and S-9, respectively. When the solution had cooled down to the room temperature, the solid products were separated and washed using deionized water and anhydrous ethanol, by the centrifugation/re-dispersion steps, and finally dried at 70 °C for 10 h. 

### 2.2. Synthesis of Modified Carbon Spheres (MCs)

MCs were synthesized by adding different kinds of surfactants with a simple one-step hydrothermal synthesis. Firstly, a 0.3 M sucrose aqueous solution was prepared as described above in Section 2.1. Secondly, the surfactants of 0.2 g hexadecyl trimethyl ammonium bromide (CTAB) (Hefei Bomei Biotechnology CO.,LTD, Hefei, China) and 0.2 g polyvinyl alcohol (PVA) (Hefei Bomei Biotechnology CO.,LTD; Mr = 74,800) were added into the sucrose aqueous solution, respectively. After vigorous stirring for 1 h, the obtained solution was transferred into a 100 mL Teflon-lined stainless steel autoclave at 160 °C for 5 h. When the solution was cooled down to room temperature, the products were collected and washed with deionized water and anhydrous ethanol, and dried at 70 °C for 10 h. Afterwards, the collected products were heat-treated at 800 °C for 2 h in a vacuum, with a heating rate of 10 °C/min. Finally, the black powders were obtained and named MCs-1 and MCs-2, respectively. For comparison, the carbon microspheres which were named MCs-0 were prepared according to a similar procedure without the addition of surfactants.

### 2.3. Material Characterization

The crystal phase of the as-prepared samples was analyzed by X-ray diffraction (XRD) measurement on a Rigaku-D/max-2400 diffractometer (JEOL Ltd., Tokyo, Japan) with Cu Kα radiation (V = 40 kV, I = 150 mA and λ = 15.406 nm) and a step interval of 0.02°, as well as a scanning speed of 8°·min^−1^ in the a scanning range of diffraction angles 2θ (10° ≤ 2θ ≤ 90°). The morphology and microstructure of the materials were examined by scanning electron microscope (SEM) of JMS-6700F (JEOL Ltd., Tokyo, Japan) operated at 5.0 KV, and the transmission electron microscope (TEM) study was carried out on a TECNI G^2^ TF20 instrument (JEOL Ltd., Tokyo, Japan). Thermogravimetric (TG) (Netzsch Ltd., Selb, Germany) analysis was performed under an argon atmosphere with a heating rate of 10 °C /min from 30 °C to 800 °C. Raman spectroscopy was used to investigate the existence of carbon.

### 2.4. Electrochemical Measurements 

The as-prepared samples were used as anode materials of lithium-ion batteries (LIBs) for electrochemical testing. The as-prepared active materials, acetylene black and polyvinylidene fluoride (PVDF) binder with a weight ratio of 8:1:1 were stirred with a solution of *N*-methyl-2-pyrrolidone (NMP) to form a homogeneous slurry first. Then, the mixture slurry was coated on a copper foil current collector and further dried at 80 °C in a vacuum for 12 h. All of the electrode loading amounts in the active materials were 0.78 mg/cm^2^, and the density of the as-prepared materials was 0.173 g/cm^3^. The thickness of the electrodes was 45 μm. The coin cell of CR2032 consisted of the prepared sample as investigated electrodes, a lithium foil was invoked as the counter-electrodes, and a Celgard 2400 microporous polypropylene film as the separator. The electrolyte was 1 M LiPF_6_ dissolved in a mixture of ethylene carbonate (EC) and 1, 2-dimethoxyethane with 1:1 volume ratio, which was carried out in a glove box. Finally, a coin cell was tested after 6 h standing. The galvanostatic charge–discharge texts of the assembled cells were collected by a LAND2001A battery test system in a voltage range of 0.005–3 V (vs Li^+^/Li). The electrochemical impedance spectra (EIS) were analyzed in a frequency range of 0.01 Hz to 105 Hz, while the disturbance amplitude was 5 mV, using a CHI660D electrochemical workstation. All electrochemical measurements were carried out at room temperature.

## 3. Results and Discussion

**Figure 1:**
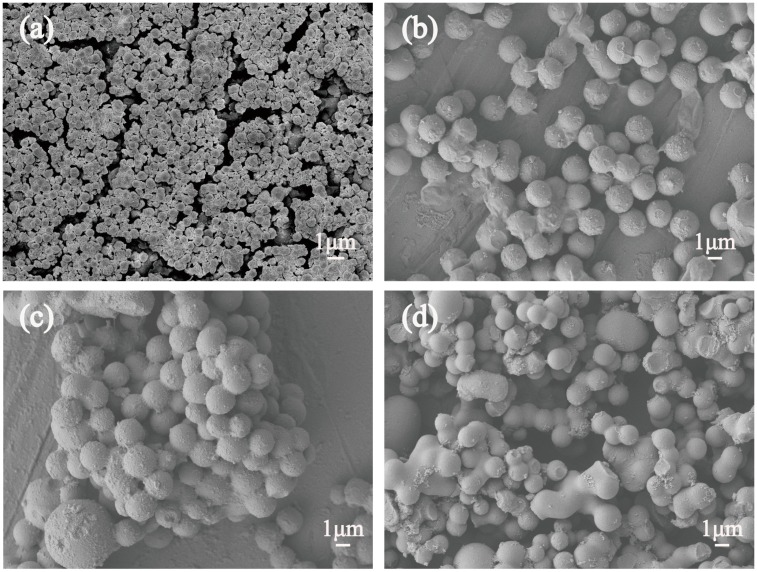
Scanning electron microscope (SEM) images of carbon spheres (Cs) with different temperatures (**a**) 160 °C (**b**) 180 °C (**c**) 200 °C (**d**) 220 °C.

The morphologies of S-1, S-2, S-3 and S-4, prepared with different temperatures and investigated by SEM, are shown in Figure 1. Once the reaction temperature was upwards of 140 °C, there was no precipitate generated; meanwhile, the color of solution was brown, and some degree of adherence appeared in the solution. The phenomenon indicates that only some aromatic clusters and oligosaccharides were generated at 140 °C, due to the experimental temperature being lower than the critical supersaturating point, which was in good agreement with the previous literature [11]. As can be noticed in Figure 1a, the products consisted of a large quantity of irregular spheres, with diameters of around 500 nm. In addition, a few spheres aggregated with each other. The distribution of the prepared Cs in Figure 1b was regular, in addition, the particle sizes of the Cs were greater than 1 μm. Furthermore the majority of the Cs seemed to be aggregated together when the temperature rose to 180 °C. As observed in Figure 1c, the Cs were made up of uniform spherical structures, with an average diameter of around 1.8 μm. However, the range of the particle size distribution of the Cs became wider as well, as the Cs were severely agglomerated together. When the temperature was enhanced to 220 °C, as shown in Figure 1d, the agglomeration of Cs occurred seriously, and the spherical structures of the products were destroyed. According to the phenomenon, some functional groups exist on the surfaces of the Cs; for instance, –OH, C=C, C–H, O=C–O, and others, through a dehydration reaction [12]. Those functional groups enhance the adsorption capacity of the surfaces, which leads to the agglomeration of the samples.

**Figure 2:**
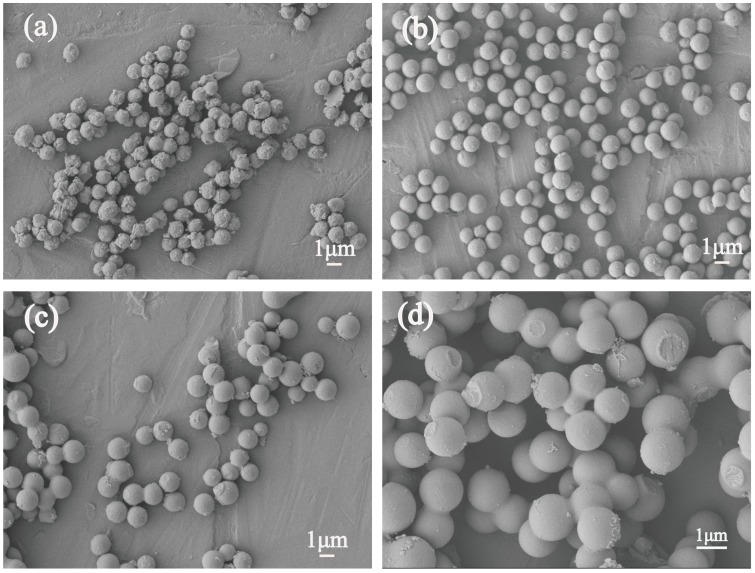
SEM images of Cs with different time periods (**a**) 5 h (**b**) 6 h (**c**) 8 h (**d**) 12 h.

Figure 2 shows SEM images of Cs with different reaction time of 5, 6, 8 and 12 h at 160 °C. When the reaction time was 4 h, as the same as that mentioned in Figure 1a showing that the samples consisted of irregular spheres with diameters of around 500 nm, as well as the Cs that had agglomerated together slightly. When the reaction time extended to 5 h, as we can see in Figure 2a, the samples were extremely close to the morphologies of the spheres, with sizes of 0.8 μm. Meanwhile, the particles separated well with each other, whereas a number of Cs small particles were absorbed on the Cs surfaces. Figure 2b illustrates that the Cs were synthesized with a good level of monodispersity, and a uniform morphology with smooth surfaces. The particle sizes of the Cs were around 1.2 μm when the reaction time was increased to 6 h. As shown in Figure 2c,d, the Cs were strongly interlinked with other particles, and the distribution of the particle size became wider, with rough surfaces when the time was prolonged to 8 and 12 h. By reason of the defective morphologies of the Cs samples synthesized in 5 h as shown in Figure 2a, whether improvements to their morphology and properties can be triggered from the addition of surfactants were carried out in the following research.

Mechanism of the formation of carbon spheres is as following: there is a large number of works related to the reactions that occurs when the saccharides are treated under supercritical water conditions at temperatures in the range of 150–350 °C [13,14,15,16,17]. The phenomenon observed in Figure 1 and Figure 2 is in consistent with the literature of [18] mentioned. In the first step, when sucrose undergoes hydrolysis at 140 °C, the orange or red of the resulting solution gives rise to an aromatic compounds and oligosaccharides; the solution has some viscosity, and no Cs can be obtained. After raising the temperature and extending the time, when the concentration of aromatic compounds in the aqueous solution reaches the critical supersaturation point, a burst nucleation process take place. Some of them grow isotropically and remain uniform after nucleation by surface diffusion or adsorption. The other parts are accumulative growth after the formation of mini-sized Cs. The formation of the Cs happens according to a nucleation growth mechanism, following the Lamer model [19], as Sun et al. [20] also proposed in relation to the hydrothermal treatment of glucose.

**Figure 3:**
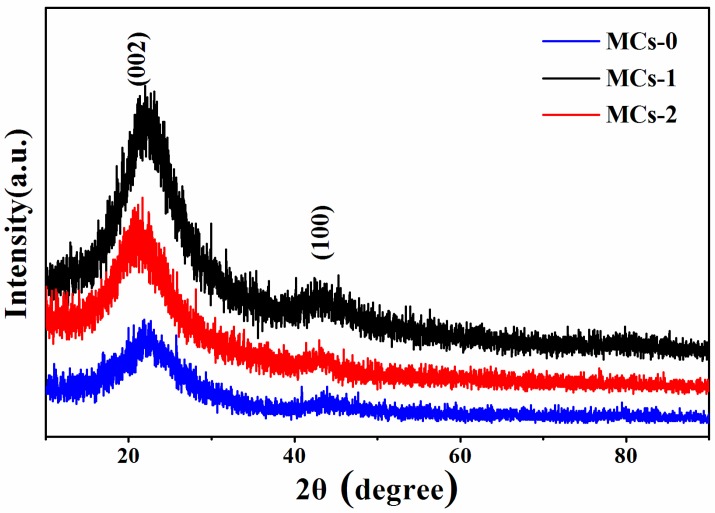
X-ray diffraction (XRD) patterns of MCs-0, MCs-1, and MCs-2.

The XRD of the modified carbon spheres (MCs) (MCs-0, MCs-1 and MCs-2 respectively representing no surfactant, CTAB or PVA) are presented in Figure 3. All the products afforded a broad reflection around 23°, and a weak reflection around 44° in the patterns, which could be attributed to the (002) and (100) reflections of the graphitic-type lattice. Therefore, the results are in accordance with the previous literature and they indicate that the Cs hold amorphous structures [21].

**Figure 4:**
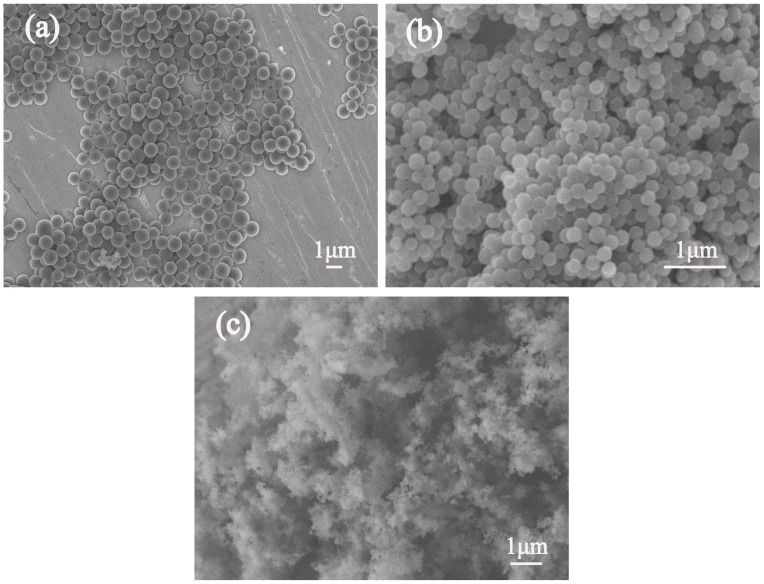
SEM images of (**a**) MCs-0, (**b**) MCs-1, and (**c**) MCs-2.

Figure 4a–c show typical SEM images of the MCs with different kinds of surfactants. It is noteworthy that the diameters of the synthesized MCs after modification with different surfactants were much smaller than those without surfactants. The synthesized MCs-1 and MCs-2 have nano-submicron structures. As shown in Figure 4a, the MCs-0 samples possess smooth surfaces, with the diameter being the range of 1.0–1.5 μm. However, the small parts of the MCs-0 are interconnected with each other. In Figure 4b, it can be seen that MCs-1, being synthesized with 0.2 g CTAB, had a uniform spherical morphology in the smooth surfaces, with the particle size being at around 200 nm. In addition, no agglomeration and abnormal growth of carbon spheres can be observed in the image. As can be seen in Figure 4c, the as-prepared MCs-2 own well nanostructure with a narrow distribution of around 70 nm, as well as the uniform spherical morphology. When the surfactant of PVA was added into the reaction system, the processes of solid nucleation, growth, and agglomeration of the Cs can be controlled, due to the chemical stability and water-solubility of PVA. Because of the good water-solubility of PVA, a further improvement in nucleation rate was achieved, and the particle sizes of Cs were reduced. Therefore, the PVA as the surfactant plays an important role in reducing particle sizes, as well as controlling the reaction processes.

**Figure 5:**
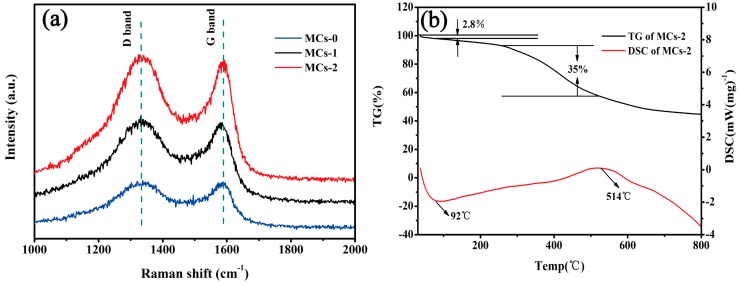
(**a**) Raman spectra of MCs-0, MCs-1, and MCs-2 (**b**) Thermogravimetry (TG)/Differential scanning calorimeter (DSC) curves of the MCs-2.

The Raman spectra in Figure 5a display two bands centered at around 1330 and 1600 cm^−1^, corresponding to the D-band (disordered structure) and the G-band (ordered graphitic structure) of the carbon, respectively. Generally, the intensity ratio of the D-band to G-band (*I_D_*/*I_G_*) is used to estimate the disorder degree of carbon materials. As the micron-sized MCs-0, for submicron-sized MCs-1 and nano-sized MCs-2 synthesized with no surfactant, CTAB, and PVA, respectively, during the preparation, the values of *I_D_*/*I_G_* increased from 1.001 and 1.039, to 1.064, respectively, indicate their highly disordered structures. The disordered structure usually showed more active sites, which is beneficial for the storage of lithium ions [22]. Figure 5b presents the thermogravimetry (TG) and the differential scanning calorimeter (DSC) results of MCs-2, which were carried out from 30 to 800 °C, with a heating rate of 10 °C·min^−1^ in an argon atmosphere. The TG analysis shows that an initial weight loss (~2.8%) of the MCs-2 occurred during the initial heating up to a temperature of 100 °C is which attributed to the water loss. As the second weight loss (~35%) occurred between 250–514 °C, this may correspond to changes in the functional groups, and the heating decomposition of unformed carbon. The reaction temperature shown in the DSC was consistent with the curve of the TG.

**Figure 6:**
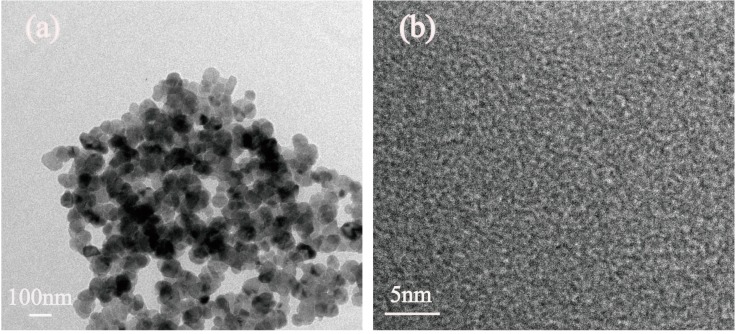
(**a**) Transmission electron microscope (TEM) (**b**) High resolution transmission electron microscopy (HRTEM) images of MCs-2.

In order to further investigate the structure of the MCs-2, TEM observations were performed and the micrographs are shown in Figure 6. As shown in Figure 6a, the MCs-2 possesses a smooth surface without any surface layer or morphology change near the surface, as well as a narrow size distribution for the average diameter, around 70 nm, which is the same result with Figure 4c. Figure 6b shows the amorphous structure of MCs-2, which is in consistent with the result from XRD analysis in Figure 3.

**Figure 7:**
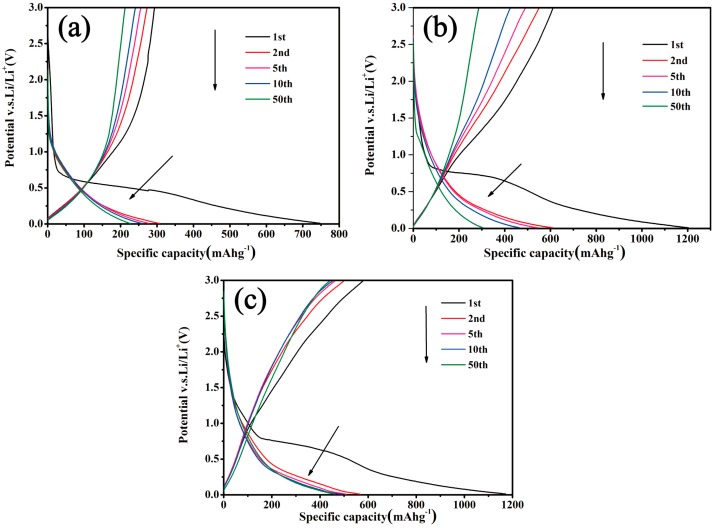
The discharge–charge curves of (**a**) MCs-0 (**b**) MCs-1, and (**c**) MCs-2 electrodes.

The electrochemical performances of the as-prepared samples were evaluated as anode materials for LIBs by CR2032 coin cells. Figure 7 illustrates that the discharge–charge profiles of the MCs electrodes; materials were measured at a current density of 100 mAg^−1^ (0.27 C) between 0.01 and 3.0 V (vs Li/Li^+^) for the 1st, 2nd, 3th, 10th, and 50th. In this cycles, the initial irreversible discharge capacities were 750, 1220, and 1180 mAhg^−1^, as well as the initial reversible discharge capacities being 308, 610, and 590 mAhg^−1^ for MCs-0, MCs-1, and MCs-2, respectively. For the initial discharge, a potential plateau of around 0.3–0.8 V corresponds to the formation of a solid electrolyte interface (SEI) layer, which results in a large irreversible loss. After 50 cycles, the discharge capacity of MCs-2 is still maintain at 470 mAhg^−1^ as shown in Figure 7c, while the discharge capacities of MCs-0 and MCs-1 attenuate from 308 mAhg^−1^ to 220 mAhg^−1^, and 610 mAhg^−1^ to 290 mAhg^−1^, respectively, showing in Figure 7a,b. Although all of the three electrode materials exhibited a capacity degradation after 50 cycles, the MCs-2 displayed the smallest capacity of decay. This is mainly because the electrochemical properties of LIBs are directly related to the particle sizes of the electrode materials. The smaller the particle are, the higher the discharge capacity is. The nanoscale of the structure, and the large specific surface areas can decrease electrode polarization, as well as can providing more migration channels for lithium ions [23]. 

**Figure 8:**
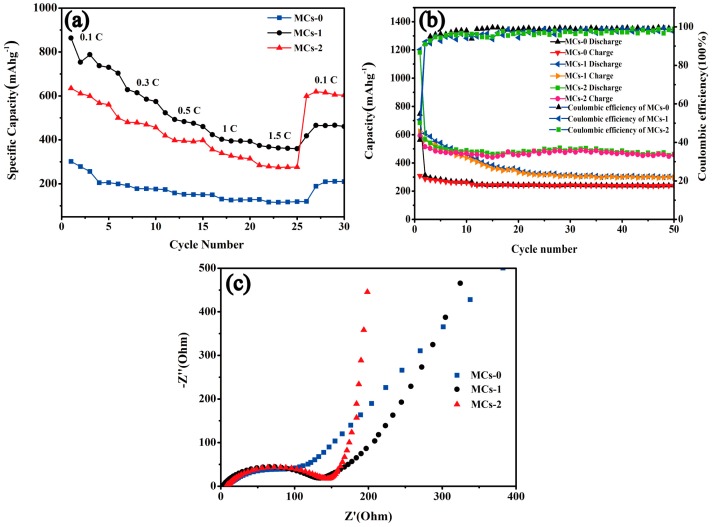
(**a**) Rate capacities of MCs-0, MCs-1, and MCs-2 electrodes; (**b**) Plots of capacity and Coulomb efficiency versus cycle number for the MCs-0, MCs-1 and MCs-2 electrodes (**c**) Nyquist plots of the MCs-0, MCs-1, and MCs-2 electrodes.

The rate capability of the electrode materials is one of the most important performance parameters for electricity storage and electric vehicle applications. Figure 8a illustrates the rate capability of the MCs electrodes materials at various rates from 0.1 to 1.5 C, in which the given capacity values are the average taken over 5 cycles. As expected, with the increase of current rates, the rate capacity decreases gradually. The obtained discharge capacity of MCs-2 is 643.9 mAhg^−1^, 500 mAgh^−1^, 419.6 mAhg^−1^, 356.2 mAhg^−1^, 284.6 mAhg^−1^ and 599.5 mAhg^−1^ as the growth of the current rates of 0.1, 0.3, 0.5, 1.0, 1.5, and 0.1 C respectively, which is more stable than that discharge capacity of MCs-0 and MCs-1, as we can see in Figure 8a directly. Therefore, the rate performance of the nano-sized MCs-2 is demonstrated to have a tremendous degree of potential, as the electrode materials appear to be more ideal than the other two. 

Figure 8b shows the charge–discharge cycling performance of the MCs electrode materials in a potential range of 0.01–3.0 V, at a current density of 100 mAg^−1^ (0.27 C). It can be clearly observed that the cycle performance curve of MCs-0 was the steadiest curve, and it showed a straight line over the 50 cycles, while the discharge capacity decreased to 230.5 mAhg^−1^ after 50 cycles. The first reversible discharge capacity of MCs-1 reached 610 mAhg^−1^, while the discharge capacity attenuated quickly, decreasing to 290 mAhg^−1^ and the coulombic efficiency was maintained at 90% after 50 cycles. In contrast, the cycle performance curve of MCs-2 was relatively steady, and the discharge capacity was maintained at about 500 mAhg^−1^ after 50 cycles, which exhibited a coulombic efficiency of higher than 95%. Therefore, the nano-sized MCs-2 delivered more excellent cycling stability than with the micron-graded MCs-0, or with the submicron-sized MCs-1 as the anode electrode of LIBs. The result reveals that the nanoscale structure of MCs-2 can shorten the lithium ion diffusion path, as well as improve the diffusion kinetics of the lithium ions.

To further reveal the reasons for the improved electrochemical performances of the MCs electrode materials, EIS of the MCs electrodes materials were conducted, as shown in Figure 8c. The Nyquist curves were composed of semicircles at high frequencies, and oblique lines at low frequencies. The semicircle stands for SEI resistance, contact resistance, and charge–transfer resistance, and the oblique lines at low frequency correspond to the diffusion resistance in internal active materials [24]. MCs-1 and MCs-2 electrode materials exhibited slightly smaller semicircle diameters than those of the MCs-0, indicating that the smaller interfacial impedances are more efficient in facilitating the charge transfer at the electrode/electrolyte interface [25]. Compared with the oblique line at low frequency, the diffusion resistance of MCs-2 was lower than MCs-0 and MCs-1 in the internal active materials. The results demonstrate that the diminished size can decrease the contact resistance and improve electrochemical performances. 

## 4. Conclusions

In summary, the Cs were successfully synthesized with sucrose through a one-step hydrothermal method in the absence of an additional treatment. Afterwards, the MCs were prepared by using a one-step hydrothermal method with different surfactants like CTAB and PVA. The samples were systematically investigated by XRD, SEM, and TEM, the nano-sized MCs modified by PVA possessed smooth surfaces, uniform spherical morphologies, and an average diameter of 70 nm. The electrochemical properties of the MCs were evaluated as anode materials for LIBs. As compared with the sub-micron-sized MCs modified with CTAB and no surfactant, the nano-sized MCs exhibited a high intial discharge capacity of 1180 mAhg^−1^, an excellent reversible discharge capacity of 470 mAhg^−1^ at 100 mAhg^−1^ (0.27 C) after 50 cycles, a remarkable rate capability (284.6 mAhg^−1^ at 1.5 C), and ideal cycling reversibility. Therefore, the nano-sized carbon materials provide an effective way to enhance electrochemical performances in LIBs, and even in practical applications involving other materials, with promising prospects.
